# Investigation of Saturation Effects in Ceramic Phosphors for Laser Lighting

**DOI:** 10.3390/ma10121407

**Published:** 2017-12-08

**Authors:** Anastasiia Krasnoshchoka, Anders Thorseth, Carsten Dam-Hansen, Dennis Dan Corell, Paul Michael Petersen, Ole Bjarlin Jensen

**Affiliations:** DTU Fotonik, Department of Photonics Engineering, Technical University of Denmark, 4000 Roskilde, Denmark; andt@fotonik.dtu.dk (A.T.); cadh@fotonik.dtu.dk (C.D.-H.); ddco@fotonik.dtu.dk (D.D.C.); pape@fotonik.dtu.dk (P.M.P.); ojen@fotonik.dtu.dk (O.B.J.)

**Keywords:** phosphor-converted, laser diode, Ce:LuAG and Eu-doped nitride, saturation

## Abstract

We report observations of saturation effects in a Ce:LuAG and Eu-doped nitride ceramic phosphor for conversion of blue laser light for white light generation. The luminous flux from the phosphors material increases linearly with the input power until saturation effects limit the conversion. It is shown that the temperature of the phosphor layer influences the saturation power level and the conversion efficiency. It is also shown that the correlated color temperature (CCT), phosphor conversion efficiency and color rendering index (CRI) are dependent both on the incident power and spot size diameter of the illumination. A phosphor conversion efficiency up to 140.8 lm/W with CRI of 89.4 was achieved. The saturation in a ceramic phosphor, when illuminated by high intensity laser diodes, is estimated to play the main role in limiting the available luminance from laser-based lighting systems.

## 1. Introduction

The gold standard for solid-state lighting (SSL), light emitting diodes (LED), have developed rapidly during the last decade and continue improving in terms of efficiency and variety of applications [1,2,3]. Recently, LEDs have become competitive with conventional light sources, such as incandescent, halogen and fluorescent lamps for general lighting applications [2]. Reducing heat generation and lowering energy dissipation, LEDs can create more energy efficient visible light for general lighting applications, as well as for specific applications [2,4]. Long lifetime and low energy consumption are some of the main factors enforcing the growth of the SSL technology.

There are two conventional methods of producing white light from LEDs, the first being the red, green, blue color model (RGB) white method, where white light is produced by mixing the output from red, green and blue LEDs. The most widely used architecture of high-brightness white LEDs is the phosphor converted white light-emitting diode (PC-LED), where a blue LED and one or more wavelength-converting phosphors are used. Due to the location of blue at the short-wavelength part of the visible spectrum, it is possible to convert blue light into green, yellow and red parts of the spectrum using phosphorescent and fluorescent materials. PC-LEDs have numerous benefits compared to the RGB white model. First, the operation principle is much easier and cheaper; second, they also do not require a lot of drivers, which makes controlling easier, and finally, blue LEDs have the highest efficiency, making the PC-LED method the most attractive in terms of efficiency.

PC-LEDs have already attracted great interest in terms of high efficiency and light quality, but despite their great commercial success, the LEDs have one significant limitation: a nonthermal droop in efficiency with increasing input current density [5,6]; thus, LED chips need to be operated at low current densities. Consequently, to ensure a high efficiency together with high flux, an increase in the number of LED chips operated at low current densities is needed. A good alternative for achieving high-luminance white light sources is using phosphor converted blue laser diodes (PC-LD) [7,8,9,10,11,12]. Replacing LEDs with laser diodes (LD) as a solid-state lighting source could be a solution for the “efficiency droop” at high input power densities. LDs operate by stimulated emission and thus they can have high efficiencies at much higher input current densities than LEDs. Above the lasing threshold, parasitic non-radiative recombination processes, including those responsible for efficiency droop in LEDs, are clamped at their rates at the lasing threshold [2,7].

PC-LDs are becoming more efficient and intense, having higher light quality at high power densities; hence, they have a tremendous potential to replace PC-LEDs for certain applications. Laser generated white light has recently been introduced for high beams by the top-line car producers, such as BMW and Audi [13,14], where the strong directionality of light generated from a small spot is of high importance. Laser–phosphor interactions have also recently been introduced in projectors, and the use of ceramic phosphor materials has proven to perform better in terms of resistance to high power densities when compared to standard silicone or epoxy based matrices [15]. However, saturation of the downconversion in the phosphors will limit the available white light from small spots. In [16,17,18], some investigations were carried out on thermal quenching of different types of phosphor materials.

In this paper, we investigate the saturation effects in a ceramic phosphor illuminated with intense blue laser light in a calibrated integrating sphere spectroradiometer setup. Using different incident laser radiant intensities, we find a general tendency in the saturation behavior of the investigated phosphors. We also investigated temperature control of the Ce:LuAG and Eu-doped nitride (Phoscera, NTK Ceratec) ceramic phosphor sample influence on the luminous flux of the downconverted blue light. We show that saturation in the ceramic phosphor is a main limiting factor to achieving high luminance light generation for lighting solutions.

## 2. Results

Firstly, the output characteristics of the blue LD were measured. The laser diode was a broad area laser diode, meaning that the beam properties of the laser will change with the appearance of higher order modes when the injection current is increased. Secondly, the beam propagation parameter, M^2^, was measured at different current levels. The measured M^2^ values vary from 1 to 2 for the fast axis and from 1.1 to 2.6 for the slow axis at the operating conditions considered in this work, confirming the high quality of the laser diode beam. The laser spot diameter in focus was approximately 148 μm.

The total spectral flux measurements of the PC-LD setup at different operating currents in the range of 0.16 A to 1 A were carried out using an integrating sphere and a spectrometer. The phosphor sample was attached to one of the ports of the integrating sphere and placed at different distances relative to the focus of the focusing lens. The first position is in focus (here, the beam size diameter is the smallest (148 μm) and, therefore, intensity is the highest). Different spot size diameter levels were obtained by moving the integrating sphere including the phosphor in steps of 2 mm, with a total of seven spot size diameter levels. The intensity of the LD decreases with increasing spot size diameter for a constant power level.

**Figure 1:**
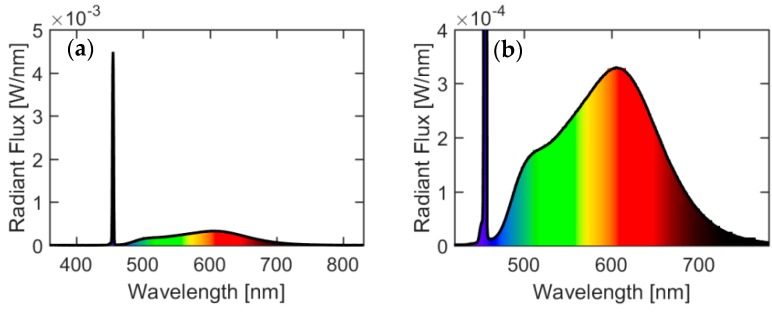
(**a**) Typical spectral power distribution for the phosphor converted blue laser diode (PC-LD) light source (**b**) Zoomed typical spectral power distribution for the PC-LD light source.

Typical spectra of the white light generated from the PC-LD light source are presented in Figure 1. The emission spectra represent a sharp blue peak with the central wavelength at λ = 455 nm and a broadband emission from the phosphor material. Calculations of the luminous flux and radiant flux emitted from the phosphor together with measurements of correlated color temperature (CCT) and color rendering index (CRI) at different power levels and laser spot size diameter were carried out from measured spectral power distributions as shown in Table 1 for two laser spot size diameters. Bearing in mind that all of the above described parameters of the output light are highly dependent on the type of the phosphor material, one could easily achieve the desired luminous flux, radiant flux, CCT and CRI parameters by using different phosphors or a different ratio and thickness of the phosphors. Data for the phosphor conversion efficiency or luminous efficacy are included in Table 1.

(1) $$\eta =\frac{\Phi_{\upsilon }}{P_{o}}$$

where Φ*_υ_* is the total luminous flux, measured in lumens (lm), emitted from the phosphor material including the blue peak from the laser diode and *P_ο_* is the input optical power from the laser diode. Looking at the spectra in Figure 1, it is clear that the spectral part coming from the phosphor material is above 465 nm. The luminous flux [19] arising from the phosphor material excluding the blue peak from the laser diode can be found as

(2) $$\Phi_{phos}=683\frac{\mathrm{lm}}{\text{W}}\int_{465\;\mathrm{nm}}^{830\;\mathrm{nm}}S(\lambda )V(\lambda )d\lambda$$

where S(λ) is the spectral power distribution of the light emitted from the phosphor material and V(λ) is the luminous efficiency function or eye sensitivity function. When the integration is performed over the wavelengths of interest, in this case from 465 nm to 830 nm, the luminous flux generated by the phosphor is calculated. Including wavelengths down to 380 nm, the total luminous flux can be calculated.

**Figure 2:**
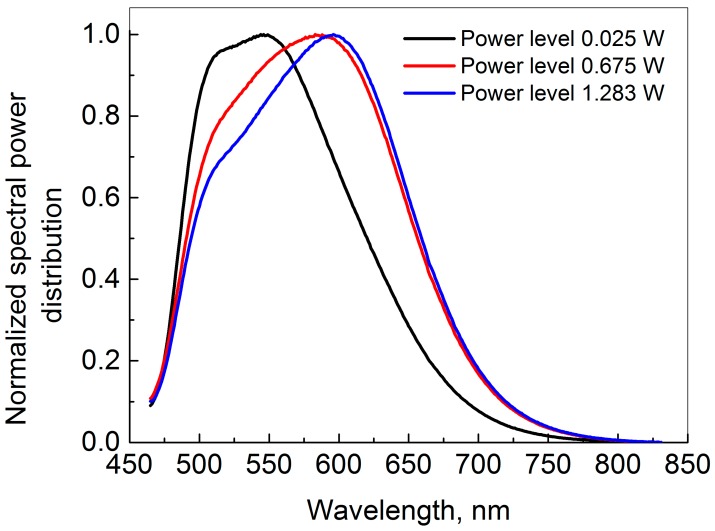
Normalized measured spectral power distribution of the phosphor emision at three different input laser powers for a laser spot diameter of 337 µm.

In Table 2, all the International Commission on Illumination CIE 1931 chromaticity *x* and *y* coordinates are shown, when the phosphor sample is illuminated by 337 µm and 585 µm laser spot size diameter. It can be seen that the chromaticity coordinates of the generated light shift dramatically at high input power levels due to saturation of the conversion. This increase in the correlated color temperature of the spectrum stems from the blue laser peaks’ relative increase with saturation. However, this is somewhat counteracted by the phosphor emission shifting towards the red, as shown by the spectra in Figure 2, where the normalized phosphorescence spectra for three different input powers at a fixed spot size of 337 µm are shown. It is seen that an increase in input laser power results in a spectral shift of the spectrum towards longer wavelengths. An increase in the centroid wavelength from 565 nm at low laser power to 587 nm at high laser power is calculated. This indicates that the green emitting phosphor (Ce:LuAG) saturates before the red emitting Eu-doped phosphor. The luminescence decay time for an Eu-doped phosphor is typically significantly longer than for a Ce-doped phosphor. Therefore, we would expect the Eu-doped phosphor to saturate at lower laser power density than the Ce-doped phosphor. In this case, the result is the opposite and this is a subject for further investigation.

**Figure 3:**
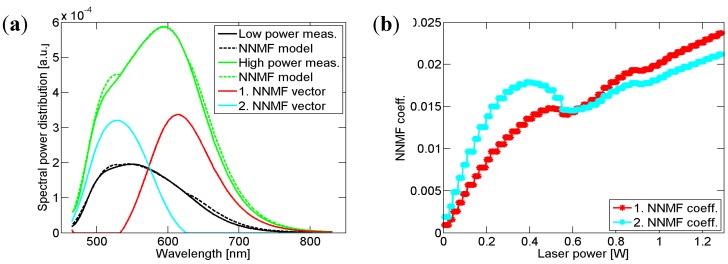
(**a**) The phosphor emission part of the spectrum estimated by the NMMF method showing the vector components and two examples at high and a low laser power of the reconstruction compared to the measured data; (**b**) the contribution of each component as a function.

The spectral power distributions of the phosphor emission measured at various laser powers have been investigated using non-negative matrix factorization (NNMF) [20]. This method takes a set of SPDs (column vectors) as input and yields an approximation to those SPDs. In our case, the method is set to generate two spectrally overlapping vectors representing the two phosphor emission peaks, and a set of coefficients—Two for each measured spectrum, representing the spectral power of each peak for a given measurement in the set. The linear combination of vectors and coefficients approximates the spectral data with typical coefficients of determination *r*^2^ of 0.995 (see Figure 3a). Studying the coefficients as a function of laser power in Figure 3b, we find that for increasing laser power the low wavelength component increases towards a certain point after which it declines and then continues with a reduced slope; the long wavelength component increases more steadily with laser power. This is consistent with the existence of two phosphor components where the low wavelength part saturates while the second only saturates to a lower degree. The correlation between the two sets of coefficients (*r*^2^ = 0.76) is an artifact of the NNMF method, where the two strongly overlapping phosphor emission peaks can only be partly separated.

**Figure 4:**
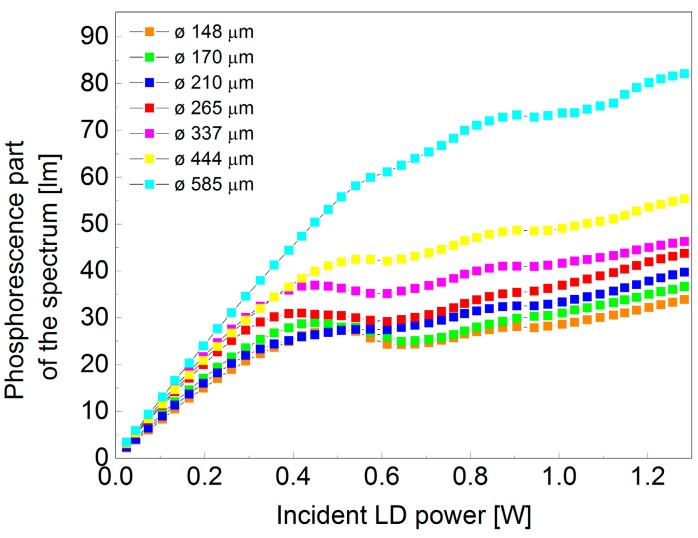
Integrated downconverted luminous flux vs input laser power at several laser spot diameters.

It is seen from Table 1 that some of the values of CCT and CRI are missing at high power. This is an indication that the light emitted from the phosphors can no longer be perceived as white. The dependence between the phosphorescence part of the spectrum (emission from the phosphor material) and input power of the blue LD was investigated to show possible saturation effects of the light emission from the excited phosphor. This dependence is illustrated in Figure 4 for seven different spot sizes diameters.

It is interesting to observe that regardless of the spot size illuminating the phosphor material, there is a general behavior that is common for all the spot sizes. Another important observation is that an increment in the incident spot size diameter will result in an increment in the luminous flux, which could be of high interest in a wide variety of lighting applications. At lower powers, the incident LD power phosphorescent part of the spectrum dependence is close to being linear and with an increment in the incident power there is a non-linear relation. We believe that saturation of the phosphor material occurs when the dependence between incident power and the phosphorescent part of the spectrum deviates from linearity. This conclusion is also in agreement with the results of CCT calculations represented in Table 1.

**Figure 5:**
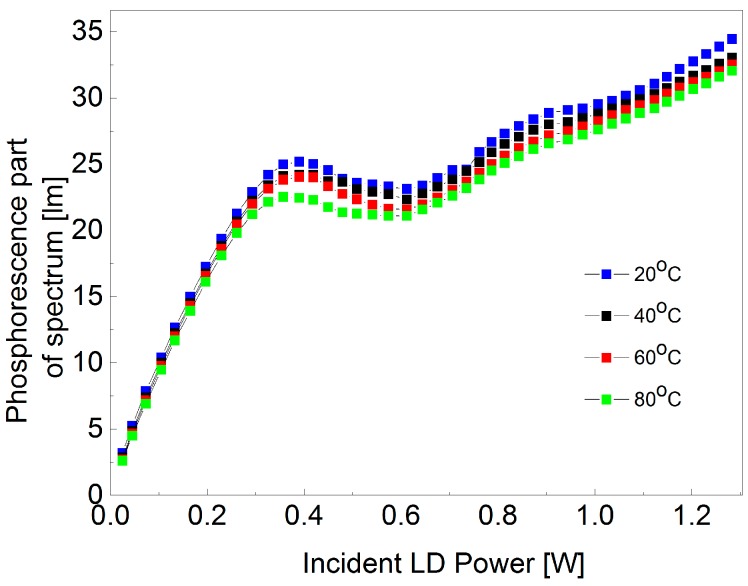
Integrated downconverted luminous flux vs input laser power at different phosphor aluminum substrate temperatures at 190 μm spot size diameter.

In order to investigate whether the saturation behavior of the ceramic phosphor depends on the phosphor temperature, we conducted a series of temperature controlled experiments of the phosphor-converted laser diodes that are illustrated in Figure 5. Here, the LD spot size diameter illuminating the phosphor material was held constant at 190 µm during the measurements.

From Figure 5, it is clearly seen that a change in temperature influences the phosphor conversion efficiency. The lower the temperature of the sample, the higher the luminous flux from the phosphorescence part of the spectrum. Furthermore, it is noticed that there is a small drop in the emission intensity after the saturation point. The small drop in converted power may be explained by a local increase in temperature at the laser spot leading to thermal quenching, when the laser power is increased. This lowers the phosphor efficiency and therefore the converted power drops. The slow increase seen at higher power may be explained by the appearance of small side lobes in the laser beam, when the power is increased. Therefore, these side lobes will have a power density below the saturation level leading to an increase in the emission. The appearance of small side lobes will effectively make the white spot a little larger and thus this is important when utilizing the light in applications requiring a small spot size. It is not possible to draw the conclusion that the only reason for saturation in the phosphor is thermal quenching, but it was shown that there is a correlation between phosphor temperature and luminous flux output. The saturation power level is only slightly dependent on the phosphor temperature.

## 3. Discussion

We have demonstrated a PC-LD white light source based on an InGaN blue laser diode and Ce:LuAG and Eu-doped nitride phosphor materials. A calibrated integrating sphere spectroradiometer setup was used to measure the total spectral flux emitted by the phosphor sample. This light source was investigated under seven different focusing conditions for the laser in order to investigate the saturation properties. One of the observations we made is that under all the different focusing conditions, Ce:LuAG and Eu-doped nitride showed the same trend in terms of luminous flux output. The next finding was a dependence between incident laser spot size and generated luminous flux. The larger the illuminated spot size diameter, the higher the amount of luminous flux, even though the input power of the laser diode was constant. We believe that this result is of high importance for a variety of lighting applications. We also defined saturation in Ce:LuAG and Eu-doped nitride phosphor as deviation from linear dependence between incident power and luminous flux output. Similar behavior of YAG:Ce^3+^ phosphor materials was found in [16,19] and explained by thermally activated luminescence quenching. To explore whether saturation of Ce:LuAG and Eu-doped nitride is connected to thermal quenching, another set of experiments was performed. The results of this led us to draw the conclusion that there is a significant relation between the operated temperature of the Ce:LuAG and Eu-doped nitride phosphor sample and the performance in terms of luminous flux output. This dependence is highly important for any application where efficient and high luminance laser phosphor converted white light is of interest. The spectral emission components of the two phosphors can be partly separated for analysis using non-negative matrix factorization. This analysis reveals that the green phosphor tends to saturate at lower power density than the red phosphor.

Nevertheless, we cannot conclude that thermal quenching is the only process that is responsible for saturation in ceramic phosphors. These effects are subjects for deeper investigations with further measurements of photoluminescence excitation spectra.

## 4. Materials and Methods

**Figure 6:**
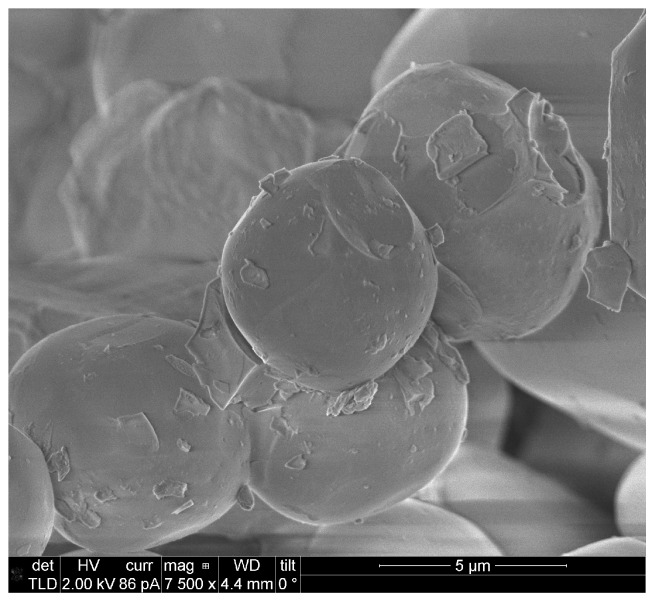
SEM images of the phosphor material used for the PC-LD light source.

A laser diode (LD) phosphor converted light source based on an InGaN laser diode and a mixture of the down-converting materials Ce:LuAG and Eu-doped nitride (Phoscera, NTK Ceratec, Tokyo, Japan) was assembled. The phosphor materials were deposited onto an aluminum substrate for good thermal handling. Scanning electron microscope (SEM) (FEI Company, Hillsboro, OR, USA) investigations of the phosphor material Ce:LuAG and Eu-doped nitride were performed and are displayed in Figure 6; in particular, the average grain diameter size of 5–10 μm and grain shape can be seen in the figure.

When the blue laser diode beam hits the phosphor sample, yellow light emitted from the sample is mixed with blue laser light resulting in high-luminance white light emission. Light hitting the substrate is reflected back into the phosphor in this configuration. The technology behind this is comparable to a white PC-LED constructed using a blue LED and phosphor material, which absorbs part of the blue light and emits light with a lower frequency, resulting in white light being emitted from the phosphor.

**Figure 7:**
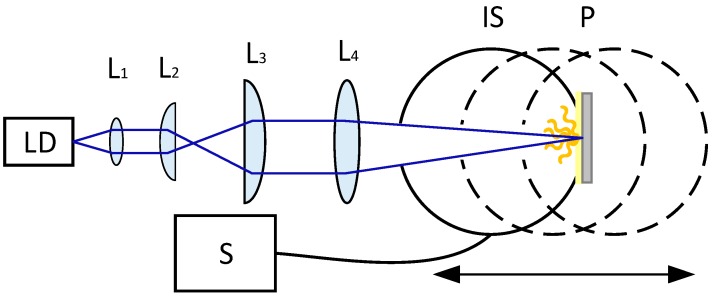
Optical scheme of the PC-LD setup. IS is an integrating sphere and S is a spectrometer. The integrating sphere, including the phosphor and temperature controled substrate, can be translated.

The configuration of the designed white light source setup for investigating saturation effects in the phosphor materials is shown in Figure 7.

The laser light emitted from the blue laser diode (λ = 455 nm) was collimated in one axis using an aspherical lens, L_1_. A cylindrical telescope consisting of two cylindrical lenses, L_2_ and L_3_, was used in order to make the LD beam circular in the focus. Following the telescope, the laser beam was focused by a focusing lens L_4_ (f = 170 mm) on the phosphor sample plate so that the white light is obtained by exciting the phosphor material with the laser diode beam.

Initially, the laser diode was characterized with respect to the output power and beam properties in order to be able to precisely estimate the radiant intensity incident on the phosphor sample. The beam parameters were characterized at all operating conditions used in the saturation measurements. A laser distance meter was used to ensure precise measurement of the focus position.

An integrating sphere spectroradiometer setup was used to measure the total spectral flux emitted in reflection by the laser illuminated sample. The focused laser beam enters the 150-mm diameter integrating sphere through a 11.3-mm diameter (1 cm^2^) entrance port and illuminates the sample placed at the 25-mm sample port at the opposite side. The sample port is equipped with a spring-loaded sample holder to hold the sample in place. The spectroradiometer (Instrument Systems, Munich, Germany) is coupled via a fiber bundle that is connected to a port at 90 degrees and baffled to both the entrance and sample port. The sphere has a BaSO_4_ coating with a high reflectance, 96%, over a wide spectral range, and the entrance port area is limited to 1 cm^2^, ensuring equal illumination of the sphere surface. The sphere spectroradiometer is calibrated using a spectral irradiance standard from an accredited laboratory, producing a known spectral irradiance at 50 cm in a specific direction; that is, a 1000-W Quartz Tungsten Halogen lamp, (Optronic Laboratories, Inc., Orlando, FL, USA) placed 50 cm from the entrance port and illuminating the sample port which during calibration is a white reference sample. The system is calibrated to the total spectral flux entering through the 1-cm^2^ entrance port in the 2π geometry. This resembles the test situation where the laser beam illuminates the sample, as opposed to the calibration in [21] where the double sphere system is calibrated using a total standard spectral flux lamp placed in the center of the spheres (4π geometry). The integrating sphere is mounted on a linear translation stage with micrometer drive for adjusting the position of the sample surface relative to the focusing lens L_4_. The distance is measured using a digital ruler with a laser cross alignment. The position of the integrating sphere including the phosphor sample is varied around the focal point of the laser beam and the actual laser spot size diameter on the sample is derived from the pre-measurements of the beam properties as a function of distance from the focusing lens L_4_. All the measurements were made in a temperature controlled laboratory at T = (25 ± 1.1) °C. The calculations for the CIE coordinates, CCT and CRI were performed according to the CIE standards [22,23].

## Figures and Tables

**Table 1:** Measured correlated color temperature (CCT), color rendering index (CRI) and phosphor conversion efficiency values when illuminated with 337 μm and 585 μm laser spot size diameter.

	337 μm Spot Size Diameter				585 μm Spot Size Diameter			
Power (W)	Power Density (W/mm^2^)	CCT (K)	CRI	Phosphor Conversion Efficiency (lm/W)	Power Density (W/mm^2^)	CCT (K)	CRI	Phosphor Conversion Efficiency (lm/W)
0.025	0.28	8772	85.1	133.8	0.09	5800	89.4	140.8
0.105	1.18	9547	86.6	121.2	0.39	5990	89.4	128.2
0.165	1.85	10,678	87.2	117.0	0.61	5986	89.5	126.8
0.229	2.57	12,088	87	112.9	0.85	6228	89.7	124.7
0.390	4.37	-	-	97.6	1.45	6740	89.5	118.1
0.706	7.92	-	-	58.9	2.63	10,285	83.9	97.4
1.148	12.87	-	-	45.2	4.27	-	-	73.3

**Table 2:** Measured CIE 1931 chromaticity coordinate values when illuminated with 337 μm and 585 μm laser spot size diameters.

Chromaticity Coordinate	Laser Spot Diameter (μm)	Power Level (W)						
		0.025	0.105	0.165	0.229	0.390	0.706	1.148
CIE *x*	337	0.2902	0.2873	0.2842	0.2809	0.2693	0.2339	0.2123
CIE *y*		0.2950	0.2830	0.2718	0.2631	0.2348	0.1576	0.1248
CIE *x*	585	0.3268	0.3238	0.3239	0.3206	0.3147	0.2949	0.2661
CIE *y*		0.3141	0.3096	0.3081	0.3023	0.2924	0.2573	0.2078
